# Polymorphisms of Immunity Genes and Susceptibility to Otitis Media in Children

**DOI:** 10.1371/journal.pone.0093930

**Published:** 2014-04-09

**Authors:** Johanna Nokso-Koivisto, Tasnee Chonmaitree, Kristofer Jennings, Reuben Matalon, Stan Block, Janak A. Patel

**Affiliations:** 1 Department of Pediatrics, University of Texas Medical Branch, Galveston, Texas, United States of America; 2 Department of Preventive Medicine and Community Health, University of Texas Medical Branch, Galveston, Texas, United States of America; 3 Kentucky Pediatric Research, Inc., Bardstown, Kentucky, United States of America; National Taiwan University, Taiwan

## Abstract

**Background:**

Acute otitis media (OM) is a common disease which often develops through complex interactions between the host, the pathogen and environmental factors. We studied single nucleotide polymorphisms (SNPs) of genes involved in innate and adaptive immunity, and other host and environmental factors for their role in OM.

**Methods:**

Using Sequenom Massarray platform, 21 SNPs were studied in 653 children from prospective (n = 202) and retrospective (n = 451) cohorts. Data were analyzed for the relationship between SNPs and upper respiratory infection (URI) frequency, risk of acute OM during URI episodes, and proneness to recurrent OM.

**Results:**

Increased risk for OM proneness was associated with CX3CR1 (Thr280Met) SNP and with a jointly interactive group of IL-10 (−1082) SNP, IL-1β (−511) wild type genotype and white race. Family history of OM proneness independently increased the risk for frequent URIs, OM occurrence during URI, and OM proneness. Additionally, IL-1β (−31) SNP was associated with increased risk for frequent URIs, but IL-10 (−592), IL-1β (−511), IL-5 (−746) and IL-8 (−251) SNPs were associated with decreased risk of URI.

**Conclusion:**

IL-1β (−31), CX3CR1 (Thr280Met), IL-10 (−1082) and IL-1β (−511) SNPs were associated with increased risk for frequent URIs or OM proneness.

## Introduction

Viral upper respiratory infection (URI) is the commonest infectious disease worldwide. Acute otitis media (OM) is a frequent complication of viral URI in children [1]. Some children are more susceptible to recurrent OM (OM-prone); the reasons for this are likely to be multifactorial, including the host genetic factors, the pathogen and environmental factors. High risk for OM proneness occurs in family clusters and specific ethnic populations, and twin and triplet studies have strongly suggested the heritability of recurrent OM [2]–[4].

Cytokines participate in the innate and adaptive immunity against infectious diseases. Many cytokines are actively induced in nasal secretions of children during viral URI, suggesting that these cytokines participate in regulation of virus-induced inflammation and recovery from infection. High levels of certain cytokines in respiratory secretions have been associated with the severity of respiratory disease [5]–[8]. We have also shown that high IL-1β levels in the nasopharynx are associated with the risk for acute OM during URI episode [9]. As single nucleotide gene polymorphisms (SNPs) of cytokine genes can modulate the production of respective cytokines, it is likely that these SNPs affect the risk for viral URI and OM. For example, we have shown an increased risk for URI as well as OM proneness with IL-6 (−174) and TNFα (−308) SNPs [10], [11]. OM proneness has been shown to be increased with IL10 (−1082), TLR4 (−299), CD14 (−159), and MBL (−54) SNPs [10], [12]–[15].

The present study aimed to investigate the role of several additional genes of innate and adaptive immunity in susceptibility to OM proneness and URI in association with host and environmental risk factors. Specifically, 21 SNPs of genes were selected based on their previously published roles in respiratory infections and OM [16], [17]. Since the study cohorts in this report were previously examined to evaluate the association of IL-1β (+3953), TNFα (−308), and IL-6 (−174) SNPs with URI and OM, data related to these three SNPs are not presented herein [10], [11].

## Methods

### Study Population

Children included in the present analysis are from two different retrospective and prospective study cohorts. The cohorts were enrolled from January 2003 through March 2007.

#### The retrospective study cohort

This cohort was enrolled to assess the genetic risk factors for OM proneness in children, age 3 yrs or older, who had pre-determined OM prone or non-prone (control) status [10]. The children were enrolled at the outpatient general pediatrics clinics and the pediatric otolaryngology clinic at University of Texas Medical Branch, Galveston, TX and at the general pediatrics clinic at Kentucky Pediatric Research Office, Bardstown, KY. Clinical data were collected by interviewing the parents and by reviewing medical charts.

#### The prospective study cohort

This cohort was followed longitudinally to investigate the incidence of URI and occurrence of AOM following URI [1]. In brief, healthy children, age 6 mos to 3 yrs were followed for one year to study the occurrences of URI and AOM. Parents informed the study personnel when the child developed URI symptoms (nasal congestion, rhinorrhea, cough and/or sore throat, with or without fever). Children were then seen by a study physician as soon as possible, and were followed for the occurrence of acute OM. At each visit, otoscopic and physical examinations were performed, and tympanometric data were recorded. Acute OM complicating URI was considered when it occurred within 28 days of the onset of URI. Acute OM was defined as 1) acute onset of symptoms, 2) signs of tympanic membrane inflammation, and 3) the presence of middle ear fluid as documented by pneumatic otoscopy and/or tympanometry.

In both cohorts, a blood sample or buccal mucosa swab was collected for DNA extraction at enrollment. Children in both cohorts were classified as OM-prone by *one* of the following criteria: 1) 3 or more episodes of OM within 6 mos.; 2) 4 or more episodes of OM within 12 mos.; 3) 6 or more episodes of OM by age 6 yrs., 4) first OM episode before age of 6 mos.; 5) history of tympanostomy tube placement for recurrent or persistent OM. Children were classified as non-OM-prone if they had only 0–1 episode of OM by age 2 yrs (with reliable documentation available in their medical records). Children with an anatomic or a physiologic defect of the ear or nasopharynx, known immunologic abnormality, or major medical conditions or treatment for chronic conditions were excluded from both cohorts.

### Ethics Statement

The study was approved by the Institutional Review Board at University of Texas Medical Branch, Galveston, TX, USA. Informed written consent was obtained from the parents of all participating children.

### Data Sharing Plan

We intend to make our original data available to interested nonaffiliated scientists. We will review each written request and honor each legitimate requests that will serve to validate our data and will advance the field provided that this request does not compromise our right to first publication of results that directly address the aims of the grant.

### SNP Assays

The whole genome DNA was extracted from the peripheral blood mononuclear cells or buccal epithelial cells and stored at −70C until further use. The specific 21 SNPs studied are shown in Table 1; C–C chemokine receptor type 5 (CCR5) −2554, CX3C chemokine receptor 1 (CX3CR1) 280, Inter-Cellular Adhesion Molecule 1 (ICAM1) K469E and I20788, Interleukin 1β (IL-1β) −31, −511, Interleukin 2 (IL-2) −330, Interleukin 5 (IL-5) −746, Interleukin 8 (IL-8) −251, Interleukin 10 (IL-10) −1082 and −592, Interleukin 12 (IL-12) −1188, Interleukin 13 (IL-13) −1055, Interleukin 18 (IL-18) 133, Mannose-binding lectin (MBL) gly54asp, RANTES −403, Transforming growth factor β (TGF-β1) −509 Toll-like receptor (TLR4) Asp299Gly and Thr399Ile, Tumor necrosis factor α (TNFα) −238, −376.

**Table 1:** Allele and genotype frequencies among 653 study subjects.

	RS number	Wild type genotype	No. withwild typegenotype	%	Heterozygouspoly-morphism	No. withheterozygouspolymorphism	%	Homozygouspolymorphism	No. withHomozygouspolymorphism	%	Total % withpolymorphism
CCR5 (−2554)	2734648	GG	300	46	GT	272	42	TT	81	12	54
CX3CR1 (Thr280Met)	3732378	GG	496	76	GA	147	22	AA	10	2	24
ICAM1 (K469E)	5498	AA	239	37	AG	283	43	GG	131	20	63
ICAM1 (20788)	885743	TT	255	39	TA	268	41	AA	130	20	61
IL-1β (−31)	1143627	AA	212	33	AG	289	44	GG	152	23	67
IL-1β (−511)	16944	GG	223	34	GA	292	45	AA	138	21	66
IL-2 (−330)	2069762	AA	388	59	AC	220	34	CC	45	7	41
IL-5 (−746)	2069812	GG	203	31	GA	307	47	AA	143	22	69
IL-8 (−251)	4073	AA	206	31	AT	267	41	TT	180	28	69
IL-10 (−1082)	1800896	TT	256	39	TC	295	45	CC	102	16	61
IL-10 (−592)	1800872	GG	291	45	GT	277	42	TT	85	13	55
IL-12B (−1188)	3212227	TT	326	50	TG	263	40	GG	64	10	50
IL-13 (−1055)	1800925	CC	357	55	CT	248	38	TT	48	7	45
IL-18 (133)	360721	GG	326	50	GC	267	41	CC	60	9	50
MBL (Gly54Asp)	1800450	CC	520	80	CT	115	17	TT	18	3	20
RANTES (−403)	2107538	CC	339	52	CT	255	39	TT	59	9	48
TGF-β1 (−509)	1800469	GG	301	47	GA	284	43	AA	68	10	53
TLR4 (Asp299Gly)	4986790	AA	595	91	AG	57	9	GG	1	0	9
TLR4 (Thr399Ile)	3732378	CC	614	94	CT	39	6	TT	0	0	6
TNFα (−238)	361525	GG	604	92	GA	47	8	AA	2	0	9
TNFα (−376)	1800750	GG	635	97	GA	18	3	AA	0	0	3

RS number = Reference single nucleotide polymorphism (SNP) number.

CCR5 = C-C chemokine receptor type 5, CX3CR1 = CX3C chemokine receptor 1, ICAM1 = Inter-Cellular Adhesion Molecule 1, IL-1β = Interleukin 1β, IL-2 = Interleukin 2, IL-5 = Interleukin 5, IL-6 = Interleukin 6, IL-8 = Interleukin 8, IL-10 Interleukin 10, IL-12 = Interleukin 12, IL13 = Interleukin 13, IL-18 = Interleukin 18, MBL = Mannose-binding lectin, TGF-β1 = Transforming growth factor β, TLR4 = Toll-like receptor, TNFα = Tumor necrosis factor α.

The SNPs were analyzed at the Center for Genotyping and Analysis of the Broad Institute of Massachusetts Institute of Technology, Cambridge, MA, using Sequenom MassARRAY platform as previously described [18].

### Statistical Analyses

An elastic net Poisson model [19] was used to model the number of URI episodes and OM occurrence during URI episodes. To model proneness, logistic regression with an elastic net penalty was used. All models included demographic factors such as breast feeding (any vs. none), day care (any vs. home care), exposure to cigarette smoke (any vs. none), family OM history (OM susceptibility in immediate family members; yes vs. no), as well as measured SNP genotypes which each had three categories ‘wild type’ (predominant genotype), ‘heterozygous polymorphism’ and ‘homozygous polymorphism’. Tuning parameters were set using out-of-sample error likelihood estimation based on 10-fold cross validation. All statistical procedures were run using libraries in the R programming environment (http://cran.r-project.org/). All of the models included IL-1β (+3953), TNFα (−308), and IL-6 (−174) SNPs, but the data related to these SNPs are not shown as they have been published previously. Additionally, since each study enrolled cohorts of differing ages, the age effect was not reported due to possible confounding.

## Results

### SNP Assay Evaluation

Altogether we had results of 21 SNPs from DNA of 747 children. However, 94 children were excluded from the analysis because of missing or inaccurate data related to OM proneness classification or environmental risk factors. In the final analysis, we used data from 653 children whose information was complete.

The distribution of the allele and genotype frequencies of 21 SNPs is shown in Table 1. The hetero- or homozygous SNP frequencies were less than 10% for TGF-β1 (−509), TLR4 (Asp299Gly), TLR4 (Thr399Ile), TNFα (−238) and TNFα (−376). The SNP frequencies exceeded 50% for CCR5 (−2554), ICAM1 (K469E), ICAM1 (20788), IL-1β (−31), IL-1β (−511), IL-5 (−746), IL-8 (−251), IL-10 (−1082), IL-10 (−592) and TGF-β1 (−509).

### Population Characteristics

The demographic and clinical characteristics of 653 study children are given in Table 2. The median age of enrollment in the retrospective cohort (4 years) was higher than in the prospective cohort (1 year) due to the enrollment criteria for each cohort. The racial/ethnicity distribution of the study subjects in the retrospective cohort reflected the general population distribution at the two study sites (Texas and Kentucky). The prospective cohort represented the distribution of general population at the Texas study site only. The retrospective cohort also had a higher number of children with OM proneness because the study using this cohort was designed to compare equivalent number of children who were OM prone or non-prone. Statistical analysis revealed no site specific effect on the results reported below.

**Table 2:** Demographic and clinical characteristics of 653 study children.

	Retrospective Study (n = 451)	%	Prospective Study (n = 202)	%
Female	190	42	99	49
Median age at enrollment (yrs)	4		1	
Race/ethnicity^a^				
White	224	50	36	18
Black	110	24	61	30
Hispanic	109	24	83	41
Asian	4	1	3	1
Biracial	4	1	19	9
Daycare attendance = yes	186	41	58	29
Breastfed^b^ = yes	176	39	98	49
Cigarette smoke exposure^c^ = yes	148	33	57	28
Family history of OM proneness^d^ = yes	211	47	96	48
OM-prone = yes	256	57	61	30*
Tympanostomy tubes	53	12	7	3

a Whites of non-Hispanic ethnicity.

b Any duration of breast feeding.

c Any duration of exposure to cigarette smoke.

d OM susceptibility in immediate family members.

### SNPs vs. Risk for OM Proneness

The risk of OM proneness was analyzed in 653 children from both the prospective and retrospective cohorts (Table 3). Increased risk for OM proneness was independently associated with CX3CR1 (Thr280Met) polymorphic genotype, family history of OM, attendance at day care, and lack of breastfeeding. Furthermore, IL-10 (−1082) SNP, IL-1B (−511) wild type genotype and white race predicted OM proneness only when analyzed together in a joint hypothesis (ie. these factors are not individually independent predictors of OM proneness). The risk of tympanostomy tube placement was positively associated with IL-2 (−330) hetero- or homozygous SNP, TGFβ1 (−509) wild type genotype, and male gender (data not shown).

**Table 3:** Logistic regression model (*R^2^* = 0.12) to predict OM proneness in 653 children.

Predictor	OR	Chi-square	P value
Family history of OM proneness = yes	2.08	19.00	<0.001
Daycare attendance = yes	1.68	8.94	0.003
Breastfed = no	1.46	6.17	0.013
White race, IL-1β (−511) and IL-10 (−1082) together^a^		11.91	0.008
Race = white^a^	1.46		
IL-1β (−511)^a^^,^ b	1.35		
IL-10 (−1082)^a^^,^ c	1.54		
CX3CR1 (Thr280Met)^d^	6.23	4.29	0.038

Only statistically significant results are shown above at P value <0.05.

a The inference for the factors of race, IL-1β (−511), and IL-10 (−1082) is a based on a joint hypothesis; thus the degrees of freedom on the chi-square (11.91) is 3, as opposed to 1 on the other inferences.

b Wild type genotype.

c Either hetero- or homozygous polymorphic genotype.

d Homozygous polymorphic genotype.

### SNPs vs. Risk for Frequent URI and OM Occurrence during URI Episode

In the prospective study cohort of 202 children, the number of URI episodes and occurrence of OM during URI episode during the one-year follow-up period were analyzed. IL-1β (−31) homozygous SNP was associated with increased risk for frequent URIs while IL-10 (−592), IL-1β (−511), IL-5 (−746) and IL-8 (−251) homozygous SNPs were associated with decreased risk (Table 4). Furthermore, decreased risk of OM occurrence during URI episodes was associated with IL-10 (−592) homozygous SNP (Table 5). Family history of OM proneness increased the risk for both frequent URIs and OM occurrence during URI episodes, while day care attendance was associated only with OM occurrence during URI episodes (Tables 4 and 5).

**Table 4:** Poisson multiple regression (*R^2^* = 0.17) model to predict the number of URI episodes in 202 children of the prospective study cohort.

Predictor	Coefficient	Chi-square	P value
**Increased risk**			
IL-1β (−31)^a^	0.42	9.00	0.003
Family history of OM proneness = yes	0.19	8.90	0.003
**Decreased risk**			
IL-10 (−592)^a^	−0.40	20.93	<0.001
IL-1β (−511)^a^	−0.62	16.81	<0.001
IL-5 (−746)^a^	−0.29	12.31	<0.001
Gender = male	−0.19	10.14	0.001
IL-8 (−251)^a^	−0.22	9.02	0.003

Only statistically significant results are shown above at P<0.005.

a homozygous polymorphic genotype.

**Table 5:** Poisson multiple regression model (*R^2^* = 0.16) to predict the number of acute OM occurrences during URI episodes in 202 children of the prospective study cohort.

Predictor	Coefficient	Chi-square	P value
**Increased risk**			
Family history of OM proneness = yes	0.30	7.66	0.005
Daycare attendance = yes	0.26	5.46	0.019
**Decreased risk**			
IL-10 (−592)^a^	−0.38	5.64	0.018

Only statistically significant results are shown above at P value <0.05.

a Homozygous polymorphic genotype.

## Discussion

We have previously shown that IL6 (−176) and TNFα (−308) SNPs are associated with frequent URI and OM proneness [10], [11]. In the present study, we further examined the role of additional 21 SNPs of immunoregulatory genes in URI and OM in children in the same study cohorts. Our data show the significant role of specific SNPs that promote or protect against URI and OM.

IL-1β is a major mediator of inflammation that plays an important role in tissue injury repair as well as in the defense against microbial pathogens. We found that IL-1β (−31) SNP was associated with increased risk for frequent URI. Chen et al have also shown increased susceptibility to chronic rhinosinusitis in association with this polymorphism [20]. On the other hand, our study showed that another functional IL-1β SNP, (−511), was associated with decreased risk for OM after URI. The reasons for the divergent results of two separate SNPs of the same cytokine gene are unknown. Watanabe et showed that IL-1β (−511) was associated with worsened systemic inflammation in sepsis [21]. In our previous study, we showed that an additional IL-1β SNP (+3954) was associated with more severe acute OM [22].

In this study, IL-10 (−592) was found to protect against both URI and occurrence of OM during URI episodes. This result is consistent with the known role of IL-10 as an anti-inflammatory cytokine; this property may result in reduced inflammation in the nasopharynx during viral infection. However, Alpert et al reported an increased risk for OM in children with IL-10 (−592) SNP during URI due to rhinovirus and respiratory syncytial virus [13]. The reasons for this discrepancy are not clear, but the subjects in different studies may have different counter-regulatory genes or differing local environmental influences that alter the susceptibility profile to disease. Furthermore, the size of our study population is much larger, and we studied the influence of genes on OM proneness to recurrent disease while Alpert et al studied the risk of OM after two specific viral upper respiratory infections. We also found that a different IL-10 (−1082) SNP was associated with OM proneness, although not with the increased frequency of URI or OM during URI episode.

IL-5 (−746) and IL-8 (−251) SNPs were found to be protective against frequent URIs. However, this effect differs from other reported effects in which they promote lower airway inflammation. For example, IL-5 (−746) SNP has been associated with worse lung functions in asthmatic children [23] and IL-8 (−251) SNP with an increased risk for bronchiolitis with respiratory syncytial virus infection [24]. It is likely that these polymorphic genes have a different influence on disease susceptibility at different body sites because different elements of host immune defenses may be required at the specific site of infection.

CX3CR1 (Thr280Met) SNP was associated with an increased risk for OM proneness. While this polymorphism is relatively rare, 9 out of 10 children with this polymorphism were OM prone which was the highest rate for any SNP. Previously, this genotype has been associated with an increased risk for lower airway complication due to infection with respiratory syncytial virus [25]. CX3CR1 is a cellular receptor on leukocytes which binds to CX3C chemoattractants. The enhanced chemotactic activity associated with the CX3CR1 (Thr280Met) may result in increased inflammation, thereby predisposing to OM.

In a subset of our subjects with tympanostomy tubes, the genotypes associated with the risk for tubes were different from the OM prone group as a whole. IL-2 (−330) hetero- or homozygous SNP, TGFβ1 (−509) wild type genotype increased the risk for tube placement. IL-2 (−330) SNP is known to increase the risk for respiratory infections [26]. TGFβ1 protein is involved in tissue healing leading to scar formation. Its role in chronic OM leading to granulation tissue in the middle ear has been demonstrated in experimental models [27]; however, the role of TGFβ1 (−509) SNP in URI and OM has not been explored.

As with many past studies, our study found that family history of OM proneness was associated with OM proneness in the child; however, we additionally showed that it also increases the risk for frequent URIs and occurrence of OM during URI. This observation strongly suggests that the URI and OM risks have strong familial pattern of aggregation which could be both due to genetic as well as environmental factors.

As with other published studies, we found that the history of breastfeeding was associated with a lower risk for OM proneness. However, we also showed that breastfeeding does not influence the risk for frequent URIs; this observation is similar to that of Chantry et al who studied U.S. children [28]. Duijts et al showed that Dutch children who were exclusively breastfed for at least 4 months had lower incidence of URIs, but protection lasted only during the first 6 mos of age [29]. Because our population was older than 6 months (median age of 1 yr), lack of effect on URI is consistent with previously published observation.

Overall, our study highlights the complex, interactive, positive and negative influences of the host immunoregulatory genes and environmental factors on susceptibility to URI and AOM. For example, IL-10 (−1082) SNP, IL-1B (−511) wild type genotype and white race predicted OM proneness only when analyzed together in a joint hypothesis (ie. these factors are not individually independent predictors). Additional statistical tools are needed to further dissect the gene, pathogen and environmental interactions. Some of the SNPs produced counterintuitive effects, suggesting that the immune regulatory genes may influence the disease manifestation through multiple intricate pathways with counter current loops of interaction.

The strength of our study is the large number of children who were studied and the wider selection of genetic SNPs that were analyzed. However, the study is limited by the relatively low frequencies of some of the SNPs in our population. Furthermore, since the predictive measures are relatively low, these models are best considered prognostic, characterizing the effective genetic component rather than predictive of any particular outcome. Currently, as none the SNP tests are available for routine clinical use, they cannot be used to guide patients for their individual risk. On the other hand, patients with OM proneness can be counselled that they may have inherited certain high risk genes as an explanation for their proneness. In addition, our study suggests that change in behavior such as increasing breastfeeding may be beneficial for disease reduction regardless of inheritance of disease susceptible genes.

## Conclusion

In addition to previously reported TNFa (−308) and IL-6 (−174) SNPs, we found additional SNPs of immunoregulatory genes which were associated with frequent URIs or OM proneness; these are CX3CR1 (Thr280Met), IL-10 (−1082) and IL-1β (−31). Additionally, IL-10 (−592), IL-1β (−511), IL-5 (−746) and IL-8 (−251) SNPs were found to have a protective role in URI. Further studies are needed to better understand the host genetic, pathogen and environmental factors in order to predict the child at risk for frequent URI and OM and to develop novel interventions for prevention and treatment.
